# Cannabidiol Toxicity Driven by Hydroxyquinone Formation

**DOI:** 10.1021/acs.chemrestox.4c00448

**Published:** 2025-01-29

**Authors:** Metzli I. Montero, Pravien S. Rajaram, Jose E. Zamora Alvarado, Kara E. McCloskey, Ryan D. Baxter, Roberto C. Andresen Eguiluz

**Affiliations:** ^†^Materials and Biomaterials Science and Engineering Graduate Program, ^‡^Chemistry Graduate Program, ^§^Chemical and Materials Engineering, ^⊥^Chemistry and Biochemistry, ^¶^Health Sciences Research Institute, University of California Merced, 5200 N. Lake Road, Merced, California 95344, United States

## Abstract

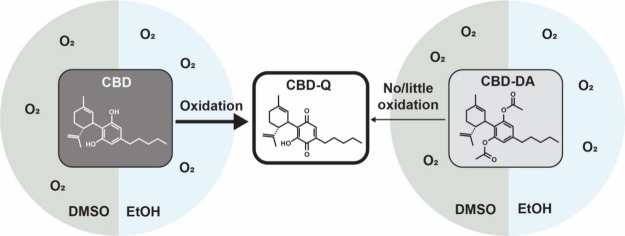

Oxidative byproducts of cannabidiol (CBD) are known to be cytotoxic. However, CBD susceptibility to oxidation and resulting toxicity dissolved in two common solvents, ethanol (EtOH) and dimethyl sulfoxide (DMSO), is seldom discussed. Furthermore, CBD products contain a wide range of concentrations, making it challenging to link general health risks associated with CBD cytotoxicity. Here, we report on the effect of CBD and CBD analogues dissolved in EtOH or DMSO at various concentrations. The cells used in these studies were human umbilical vascular endothelial cells (HUVECs). Our findings show significant CBD oxidation to cannabidiol-quinone (CBD-Q) and subsequent cytotoxicity, occurring at 10 μM concentration, regardless of the solution delivery vehicle. Moreover, a new analogue of CBD, cannabidiol-diacetate (CBD-DA), exhibits significantly more stability and reduced toxicity compared with CBD or CBD-Q, respectively. This knowledge is important for determining concentration-dependent health risks of complex cannabinoid mixtures and establishing legal limits.

Cannabis has been the source of social and political debate for decades, perpetuated by its contradictory therapeutic and detrimental effects on human health. A few therapeutic uses include the treatment of mental health disorders, chronic pain management, cancer treatment, and alleviating chemotherapy-induced nausea.^1−5^ However, detrimental effects include increased susceptibility to respiratory diseases and adverse cardiovascular events, such as bronchitis and stroke.^6−10^ As a result of conflicting results, worldwide policies regulating cannabis use are highly varied. Research aiming to understand the efficacy and safety of the over 550 chemicals that have been identified in the plant is growing.^11,12^

One of the chemicals isolated from cannabis is cannabidiol (CBD), a nonpsychoactive phytocannabinoid with ongoing investigations in various pharmacological contexts, and one FDA-approved drug already in US markets, EPIDIOLEX, used for treating two types of epilepsy disorders.^13,14^ Current studies are also examining CBD’s pharmacological potential to treat pain and cancer, including its ability to inhibit angiogenesis, attenuate the inflammatory response, and regulate vasodilation and vasoconstriction.^15−24^ To investigate CBD use, researchers have utilized both *in vivo* animal and *in vitro* human cell culture models. However, drug dosage, route of administration, and individual clinical history within specific contexts all play critical roles in the efficacy or harm after administration or consumption, complicating quantitative outcomes assessments. Additionally, there is more variety in drug source, drug vehicle, and sample preparation between current studies, making it even more difficult to compare results.^25^

To address these ongoing challenges, we present evidence supporting the hypothesis that the toxicity of oxidized cannabinoids contributes to the adverse health effects associated with cannabis use. To test this hypothesis, we first demonstrate that CBD oxidizes to form cannabidiol-quinone (CBD-Q) in a dose-dependent manner in two frequently used solvents in cell culture: ethanol (EtOH) and dimethyl sulfoxide (DMSO). Then, we used these two solvents as drug vehicles for CBD, CBD-Q, and a more stable cannabidiol, cannabidiol-diacetate (CBD-DA), in cytotoxicity studies involving human umbilical vein endothelial cells (HUVECs), a cell type used to model intravenous drug delivery. We compared the effects of both drug vehicle and drug dosage on cell viability. Following a protocol from pre-existing literature, we tested two dosages: 1 and 10 μM.^26,27^ Controls and 6 μM dosage results are included in the Supporting Information (Figure S8 and Figure S9).

With this study, we confirmed that CBD-Q was more toxic than CBD and CBD-DA, with all analogues presenting concentration-dependent toxicity. Furthermore, our findings support other reports showing that above a critical concentration (as is the case for 10 μM)^26^ leads to the induction of cellular death. In contrast, we also see proliferative effects at lower concentrations (1 μM), suggesting cell protectivity.^26^ With this investigation, we emphasize CBD’s instability, how this instability may affect toxicity studies, the importance of detailing drug vehicle storage and preparation, and the need for continuing comparative studies involving the impact of drug vehicles on CBD and its analogues.

The stability of CBD was quantified and compared with two additional CBD analogues: an isolated cannabidiol quinone, denoted as CBD-Q, and a synthesized cannabidiol-diacetate, denoted as CBD-DA (SI S1 Synthesis Procedures). The CBD-DA control was exclusively synthesized and tested to address the cytotoxic effects of CBD degradation to CBD-Q. To prevent sample degradation of CBD, the CBD compounds and its analogues were stored under a 99.9% argon atmosphere at −20 °C for up to one month prior to cell culture experiments. Upon retrieval, they were dissolved in DMSO or EtOH and used immediately.^28^ For investigating the long-term stability of CBD in solution, CBD in DMSO and CBD in EtOH solutions were also stored in the dark for one month at 4 °C, not purged, and kept within a 1 mL parafilm-sealed centrifuge tube to replicate common storage practices, and then characterized using mass spectroscopy (SI S2. Mass Spectrometry). After one month, mass spectroscopy revealed that both the DMSO and EtOH samples displayed a decrease in the relative abundance of CBD. While the relative abundance of CBD in EtOH decreased with 20% remaining and 80% converted to CBD-Q, CBD in DMSO solution completely degraded with no trace of CBD after one month with 100% converted to CBD-Q (Figure 1a).

**Figure 1:**
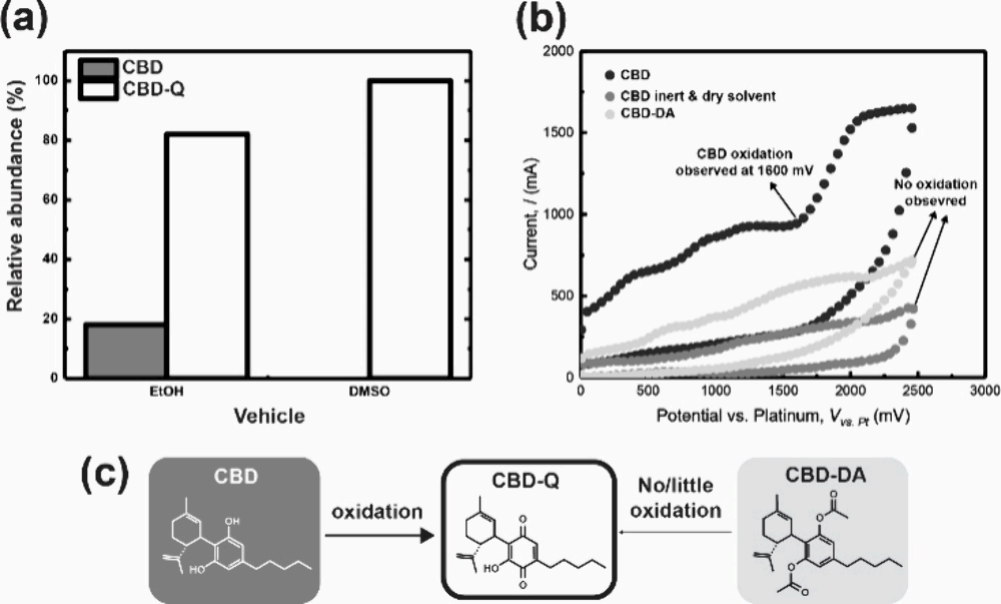
(a-c). Stability of CBD, CBD-Q, and CBD-DA. (a) Relative abundance of CBD and CBD-Q, after being dissolved in DMSO or EtOH and stored in the dark for one month at 4 °C. (b) Cyclic voltammogram displaying the oxidation segments for CBD, CBD in inert and dry solvent, and CBD-DA. (c) CBD oxidation to CBD-Q and lack of oxidation of CBD-DA to CBD-Q.

The 100% loss of CBD in DMSO and the relative abundance of CBD-Q in both samples indicate that the oxidation of CBD to CBD-Q is greater in DMSO. Cyclic voltammetry was performed to assess the oxidative susceptibility of CBD and CBD-DA (SI S3. Cyclic voltammetry procedures). The cyclic voltammogram (Figure 1b) shows a distinct oxidation potential for CBD at approximately 1600 mV, indicating its high oxidative susceptibility. When the cyclic voltammetry is run under inert (oxygen-free) and dry solvent conditions, the oxidation of CBD is not observed (Figure 1b and Figure S7a). This supports the conclusion that storing CBD in an oxygen-rich environment leads to oxidation products like CBD-Q. Thus, for oxygen-sensitive compounds, the choice of solvent or drug vehicle matters, especially when we consider existing literature that has reported EtOH to have a higher oxygen solubility than DMSO.^29^ Additionally, factors such as storage temperature and light can impact the production of oxidation products from CBD, making CBD’s integrity a challenge to control outside of dry, inert conditions.

For cytotoxicity assays, we followed previously described protocols.^26,27^ With special consideration to how CBD may degrade into CBD-Q in oxygen-rich solutions over time, we used CBD analogue samples for cell culture studies immediately upon retrieval from storage in −20 °C argon. Then, on the same day of cell experiments, we diluted CBD analogues using EtOH or DMSO into EGM-2 (Lonza, CC-3162), yielding a final working concentration of 1, 6, and 10 μM. The CBD analogue-loaded EGM-2 media was kept in the dark, at standard laboratory conditions, in a parafilm-sealed centrifuge tube for less than 1 h before being used to treat HUVECs at the working concentrations for 24 h. Afterward, cells were stained with calcein-AM (Invitrogen, C3099) and imaged at 10x using a fluorescent microscope. Images were then analyzed using FIJI with predetermined size exclusion thresholds used for cell counting, followed by a student *t*-test and 3-way ANOVA (S6 Statistical Analysis) to determine statistically significant differences between conditions.^30^ Detailed methods can be found in SI S4. Cell Culture and S5. Quantitative analysis of cytotoxicity and S6. Statistical analysis.

At the lowest 1 μM concentration of CBD, CBD-Q, and CBD-DA, all cannabinoid analogues yielded a slight decrease in the average live cell count compared to the solution control (Figure 2a and 2b). Still, this decrease was only statistically significant in the EtOH control compared with the 1 μM CBD-DA (Figure 2b). Additionally, the endothelial cell morphologies and confluency appears slightly different in several conditions compared to the controls, but cell morphology differences are likely related to confluency in each image.

**Figure 2:**
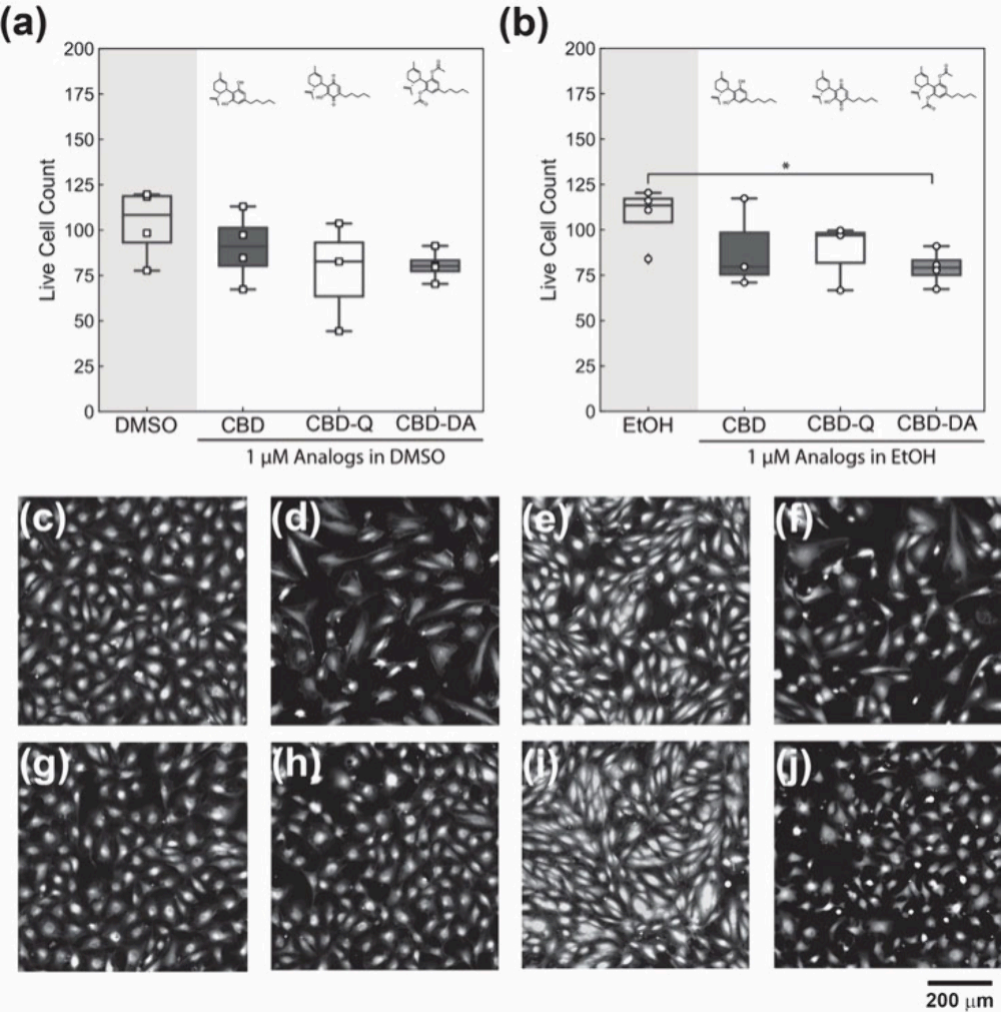
HUVEC viability after 24-h exposure to CBD, CBD-Q, and CBD-DA at 1 μM in (a) DMSO as a vehicle and (b) EtOH as a vehicle. Boxplots include the averaged cell counts of each external replicate, represented by the data points, and whiskers representing the upper and lower quartile. Micrographs of HUVECs with live stain calcein-AM exposed to (c) only DMSO (*N* = 4), (d) CBD in DMSO (*N* = 4), (e) CBD-Q in DMSO (*N* = 3), (f) CBD-DA in DMSO (*N* = 3), (g) only EtOH (*N* = 4), (h) CBD in EtOH (*N* = 4), (i) CBD-Q in EtOH (*N* = 3), and (j) CBD DA in EtOH (*N* = 3). Scale bar = 200 μm.

Because the toxicity of the oxidized metabolites may not directly correlate to the oxidation potentials, it is essential to establish which cannabinoids yield products posing the most significant risk for adverse health effects.

At the highest concentration, 10 μM, as hypothesized, all conditions displayed a significant decrease in the average live cell count compared to 1 μM conditions, with the CBD and CBD-Q in DMSO and EtOH exhibiting the largest cytotoxicities. Additionally, for the CBD, CBD-Q, and CBD-DA in DMSO, a student *t*-test analysis indicated all of the 10 μM DMSO conditions possessed an averaged live cell count that was significantly lower than the control (Figure 3a). CBD-DA exhibited the highest cell survivability of all the 10 μM analogue conditions, with cell counts at 75 ± 2 per field of view. In contrast, CBD and CBD-Q measured 60 ± 10 and 30 ± 9 cell counts per field of view, respectively. Moreover, 10 μM CBD, CBD-Q, and CBD-DA treatment groups were not statistically different, demonstrating that at 10 μM, all CBD analogue conditions displayed significant cytotoxicity.

**Figure 3:**
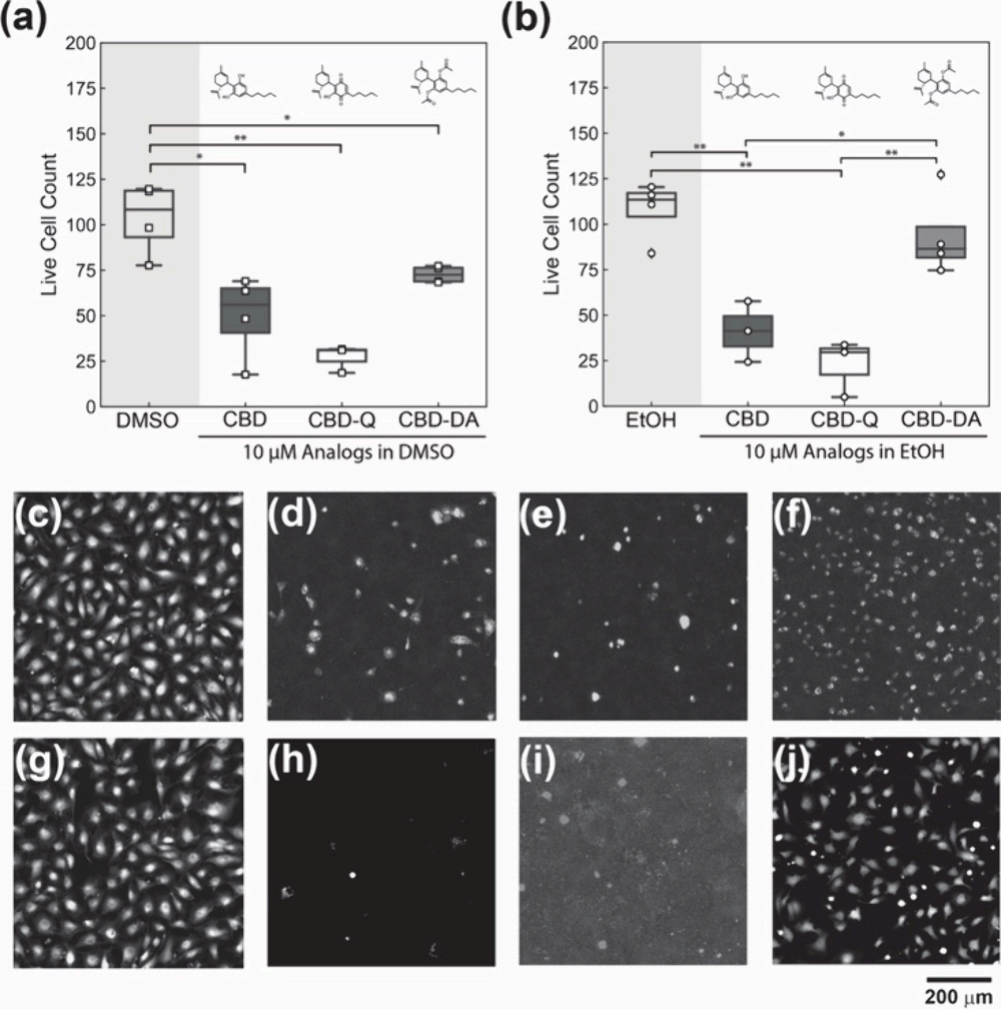
HUVEC viability after 24-h exposure to CBD, CBD-Q, and CBD-DA at 10 μM in (a) DMSO as a vehicle and (b) EtOH as a vehicle. Micrographs of HUVECs with live stain calcein-AM exposed to (c) only DMSO, (d) CBD in DMSO, (e) CBD-Q in DMSO, (f) CBD-DA in DMSO, (g) only EtOH, (h) CBD in EtOH, (i) CBD-Q in EtOH, and (j) CBD DA in EtOH. Scale bar = 200 μm.

The analogues delivered in the EtOH vehicle exhibited similar trends, with CBD and CBD-Q in EtOH treatments significantly decreasing cell survivability relative to the EtOH control. However, the average live cell count of CBD-DA in EtOH was only slightly lower than the EtOH control, and this decrease was not statistically significant. When comparing the CBD, DBD-Q, and CBD-DA only (not against the control), the cells treated with CBD-DA in EtOH possessed a significantly greater average live cell count per field of view and considerably higher survivability than CBD and CBD-Q in EtOH. The decrease in cell viability in CBD and CBD-Q in both DMSO and EtOH is easily observed visually (Figure 3 c-j), where a complete eradication of the cell monolayer and overall reduction of cell attachment is evident while the CBD-DA micrographs from both DMSO and EtOH solution treatments (Figure 3f and 3j) show that significantly more live cells remain. Still, some differences in cell morphology can be seen when comparing control samples and CBD-DA micrographs. The calcein-AM stain appeared more continuous and localized around the center of the cell body in the controls, while in the CBD-DA micrographs, one could observe a slight speckling of the fluorescent signal, suggesting a reduction of fluorescent calcein-AM production, which could result from cell death. Regardless, the presence of the cell within the micrograph indicates that the cells remained attached after washing with PBS and before imaging after 24 h. We also observed some edge-effect-dependent toxicity (not shown), where cells at the well edges experienced a greater cell death compared with those at the center of the well. This greater cell death might have been due to the lower cell density at the well-edges, suggesting toxicity also to be cell density-dependent. To mitigate this, all images presented and analyzed were taken at the approximate center of the well.

Taking these considerations into account, our results demonstrate that CBD in DMSO or EtOH is unstable and has the potential to degrade into CBD-Q, a significantly more cytotoxic analogue. Even though EtOH exhibits a higher oxygen solubility, cytotoxic cell assays reveal similar results in the effects of CBD analogues, regardless of vehicle, on HUVEC monolayers.

Based on our observations from cyclic voltammetry and cytotoxic assays, we conclude that CBD-Q is a potential culprit for decreased cell survivability. Within any given cell assay, solvents are exposed to some degree of light, oxygen, and/or heat either during the preparation, storage, or experimental process. Since CBD is sensitive to oxygen, degradability can cause experimental results to be unclear as to whether CBD is toxic or whether byproducts, like CBD-Q, are the true source of toxicity. To maintain sample purity and reliability of subsequent cytotoxicity assays, we recommend a few sample storage methods to minimize the oxidation of CBD compound. In our case, CBD was kept as a crystalline powder, where the powder was pumped and purged to remove air and moisture and then kept in a dark, inert argon environment. Additionally, we used prepared analogue solvents immediately, as preliminary results had revealed increased cytotoxicity depending on the length of analogue-solvent storage. This suggests that future cytotoxicity studies on CBD must distinguish whether CBD’s toxicity arises from byproducts produced by CBD or due to the compound’s inherent toxicity. The sensitivity of our samples also highlights the importance of detailing the exact storage conditions and durations of their analogue-solvent solutions.

We conclude by emphasizing how new CBD analogues, such as the newly synthesized CBD-DA, can be designed with improved oxidation resistance and reduced cytotoxicity.

Understanding CBD analogue stability in various environments is vital for cell applications and further studies in drug delivery. Here, we demonstrate that CBD degrades into a cytotoxic compound, CBD-Q, that is increasingly toxic to cells when exposed to oxygen-rich environments. Therefore, we demonstrate the importance of limiting a sample’s oxygen exposure, detailing sample preparation and storage within the literature, and verifying sample purity before usage to compare current and future toxicity studies on CBD and related analogues.

## Supporting Information Available

Details on experimental approaches and additional schematics and data are provided as a PDF containing: **S1.** Synthesis Procedures. Figure S1. ^1^H NMR of cannabidiol quinone. Figure S2. ^13^C NMR of cannabidiol quinone. Figure S3: ^1^H NMR of Diacetyl Cannabidiol. Figure S4: ^13^C NMR of Diacetyl Cannabidiol. **S2.** Mass spectrometry. Figure S5: Mass spectrogram of XYZ. Figure S6: Mass spectrogram of XYZ. **S3.** Cyclic voltammetry procedures. Figure S7: Cyclic voltammograms of CBD in rich and poor oxygen environments. **S4.** Cell Culture **S5.** Quantitative analysis of cytotoxicity Figure S8: HUVEC viability after 24-h exposure to controls Figure S9: HUVEC viability after 24-h exposure to CBD, CBD-Q, and CBD-DA at 6 μM **S6.** Statistical analysis. Table S1: Levene’s Test of Equality of Error Variances Table S2:3-way ANOVA Analysis Results (PDF)

## ABBREVIATIONS

CBD: cannabidiol
CBD-Q: cannabidiol quinone
CBD-DA: cannabidiol diacetate
EtOH: ethanol
DMSO: dimethyl sulfoxide
PBS: phosphate buffer saline.
