# Supplementary material for: Cannabidiol Toxicity Driven by Hydroxyquinone Formation
Source: Chem Res Toxicol. 2025 Jan 29;38(2):231–5. doi: 10.1021/acs.chemrestox.4c00448 (PMC11837211; doi:10.1021/acs.chemrestox.4c00448)
Supplement: Supplementary file 1 — tx4c00448_si_001.pdf [file tx4c00448_si_001.pdf]

Supporting Information

Cannabidiol Toxicity Driven by Hydroxyquinone Formation

Metzli I. Montero[1‡], Pravien S. Rajaram[2‡], Jose E. Zamora Alvarado[1], Kara E. McCloskey[3,5], Ryan D. Baxter[4]*, Roberto C. Andresen Eguiluz[3,5]*

[1]Materials and Biomaterials Science and Engineering Graduate Program, [2]Chemistry Graduate Program, [3]Chemical and Materials Engineering, [4]Chemistry and Biochemistry, [5]Health Sciences Research Institute, University of California, Merced, 5200 N. Lake Road, CA 95344, USA

[‡]Contributed equally

***Corresponding authors:**

* Ryan D. Baxter: rbaxter@ucmerced.edu

* Roberto C. Andresen Eguiluz: randreseneguiluz@ucmerced.edu

S1. Synthesis Procedures.

Figure S1. ^{1}H NMR of cannabidiol quinone.

Figure S2. ^{13}C NMR of cannabidiol quinone.

Figure S3: ^{1}H NMR of Diacetyl Cannabidiol.

Figure S4: ^{13}C NMR of Diacetyl Cannabidiol.

S2. Mass spectrometry.

Figure S5: Mass spectrogram of XYZ.

Figure S6: Mass spectrogram of XYZ.

S3. Cyclic voltammetry procedures.

Figure S7: Cyclic voltammograms of CBD in rich and poor oxygen environments.

S4. Cell Culture

S5. Quantitative analysis of cytotoxicity

Figure S8: HUVEC viability after 24-hr exposure to controls

Figure S9: HUVEC viability after 24-hr exposure to CBD, CBD-Q, and CBD-DA at 6 μM

S6. Statistical analysis

Table S1: Levene's Test of Equality of Error Variances

Table S2: 3-way ANOVA Analysis Results

S1. Synthesis Procedures

1.1 Synthesis of desired (1'R,6'R)-6-hydroxy-3'-methyl-4-pentyl-6'-(prop-1-en-2-yl)-[1,1'-bi(cyclohexane)]-2',3,6-triene-2,5-dione (***Cannabidiol Quinone***)

2-[(1R,6R)-6-Isopropenyl-3-methylcyclohex-2-en-1-yl]-5-pentylbenzene-1,3-diol (CBD) (200 mg, 0.63 mmol), was dissolved in 2.5 mL of Acetone. Then, potassium carbonate (K2CO3) (219.4 mg, 1.59 mmol) was added to this solution. After that, the reaction was stirred for 24 hrs at room temperature. After 24 hrs the reaction mixture was deep yellow color. TLC confirmed the completion of the reaction, and the yellow crude was subsequently purified by column chromatography with hexane/ethyl acetate (v/v = 2/1), to afford (1'R,6'R)-6-hydroxy-3'-methyl-4-pentyl-6'-(prop-1-en-2-yl)-[1,1'-bi(cyclohexane)]-2',3,6-triene-2,5-dione (23 mg, 11 % yield). ^{1}H NMR (500 MHz, cdcl$_3$) δ 6.99 (s, 1H), 6.44 – 6.34 (m, 1H), 5.14 (s, 1H), 4.58 – 4.50 (m, 2H), 3.79 – 3.67 (m, 1H), 2.76 (ddd, J = 13.4, 10.8, 2.9 Hz, 1H), 2.40 (tt, J = 7.3, 1.7 Hz, 2H), 2.22 (ddd, J = 18.0, 9.0, 3.4 Hz, 1H), 2.00 (dd, J = 17.4, 5.6 Hz, 1H), 1.77 (ddt, J = 12.9, 5.2, 2.3 Hz, 1H), 1.68 (d, J = 2.6 Hz, 3H), 1.60 (s, 1H), 1.54 – 1.48 (m, 2H), 1.36 – 1.31 (m, 4H), 1.25 (s, 3H), 0.92 – 0.88 (m, 3H). ^{13}C NMR (126 MHz, cdcl$_3$) δ 187.16, 184.05, 151.27, 148.40, 144.52, 134.67, 134.00, 122.75, 122.35, 110.65, 44.67, 35.74, 31.36, 30.45, 29.69, 28.82, 28.13, 27.12, 23.42, 22.34, 18.68, 13.90. IR (FTIR): 829, 1052, 1173, 1377, 1584, 1704, 2856, 2927, 3444 cm-1. MS (ESI): m/z calcd. for $C_{21}H_{28}O_3$ $(M+H)^+$ 329.2111, found 329.2111.

1.2 Synthesis of desired (1'R,2'R)-5'-methyl-4-pentyl-2'-(prop-1-en-2-yl)-1',2',3',4'-tetrahydro-[1,1'-biphenyl]-2,6-diyl diacetate (***Diacetyl Cannabidiol***)

2-[(1R,6R)-6-Isopropenyl-3-methylcyclohex-2-en-1-yl]-5-pentylbenzene-1,3-diol (**CBD**) (942 mg, 3 mmol), was dissolved in 5.5 mL of pyridine. Then, to this solution, acetic anhydride (5.4 g, 52 mmol) was added. After that, the reaction was stirred for 24 hrs at room temperature. After 24 hrs the reaction mixture was confirmed by TLC for completion, and the crude was subsequently purified by column chromatography with hexane/ethyl acetate (v/v = 10/1), to afford (1'R,2'R)-5'-methyl-4-pentyl-2'-(prop-1-en-2-yl)-1',2',3',4'-tetrahydro-[1,1'-biphenyl]-2,6-diyl diacetate (1.18 g, 99 % yield). ^{1}H NMR (500 MHz, cdcl$_3$) δ 6.72 (s, 2H), 5.21 (d, J = 2.7 Hz, 1H), 4.56 (t, J = 1.8 Hz, 1H), 4.47 (d, J = 2.2 Hz, 1H), 3.52 (ddt, J = 10.9, 4.5, 2.2 Hz, 1H), 2.66 (ddd, J = 13.4, 10.6, 3.1 Hz, 1H), 2.56 (dd, J = 9.0, 6.7 Hz, 2H), 2.27 – 2.12 (m, 7H), 2.04 (dd, J = 17.7, 5.3 Hz, 1H), 1.85 – 1.70 (m, 2H), 1.68 (s, 3H), 1.59 (s, 5H), 1.31 (qd, J = 7.8, 6.9, 4.3 Hz, 4H), 0.89 (t, J = 6.8 Hz, 3H). ^{13}C NMR (126 MHz, cdcl$_3$) δ 149.61, 147.82, 142.02, 135.75, 132.89, 130.63, 125.88, 124.52, 111.06, 45.61, 38.41, 35.19, 31.47, 30.43, 30.30, 28.68, 23.44, 22.46, 20.95, 19.61, 14.01. IR (FTIR):886, 1032, 1183, 1366, 1770, 2875, 2972 cm-1 MS (ESI): m/z calcd. for $C_{25}H_{34}O_4$ $(M+H)^+$ 399.2529, found 399.2529.

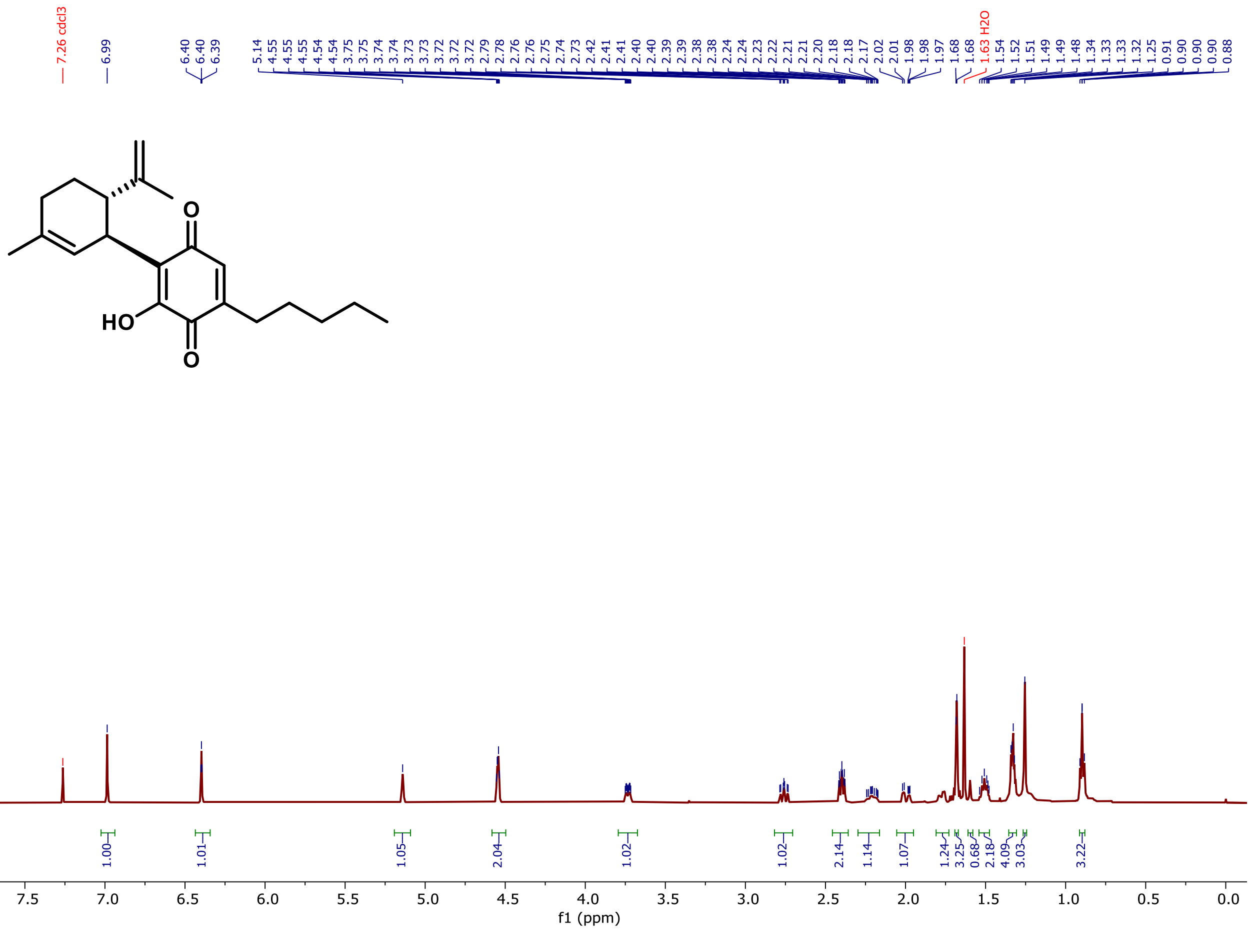

Figure S1. ^{1}H NMR of cannabidiol quinone.

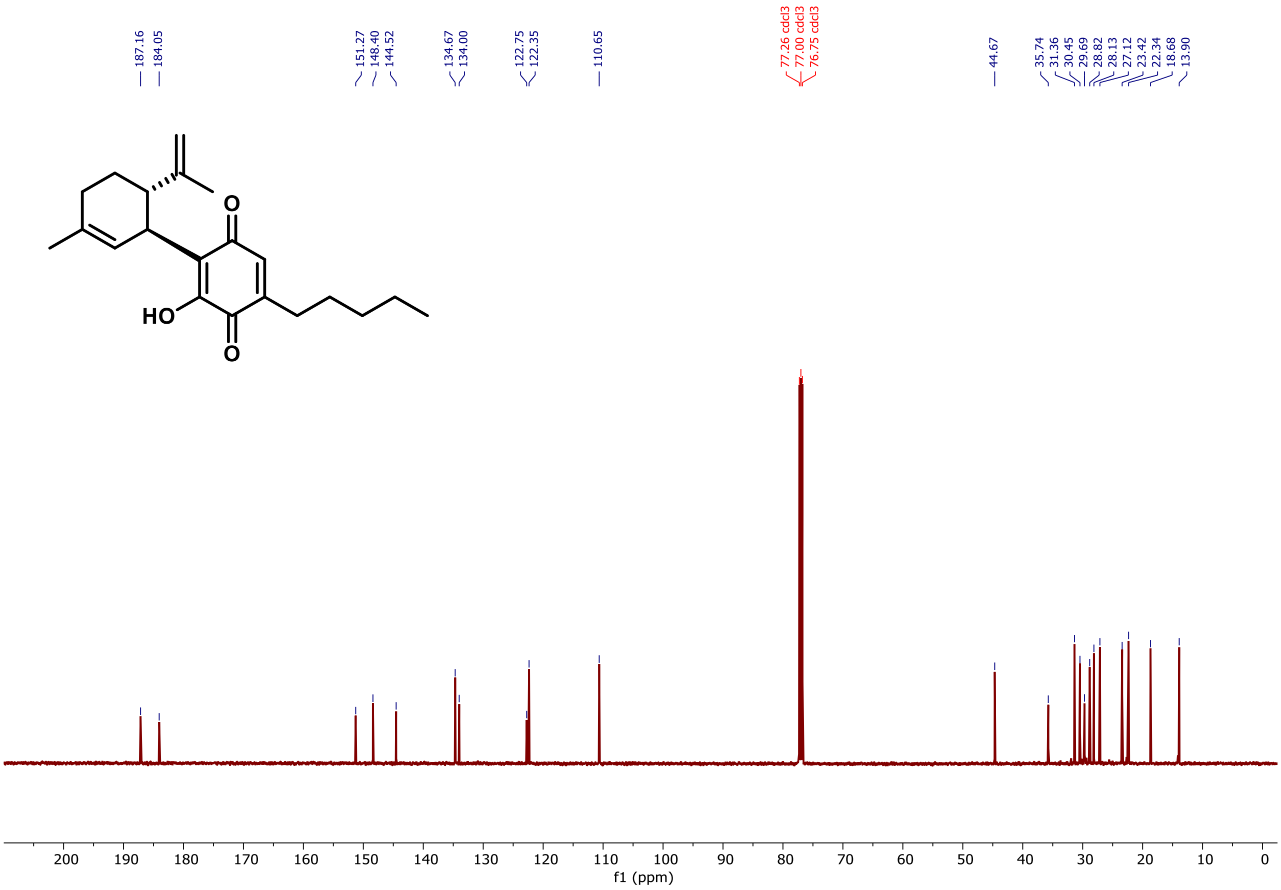

Figure S2: ^{13}C NMR of cannabidiol quinone.

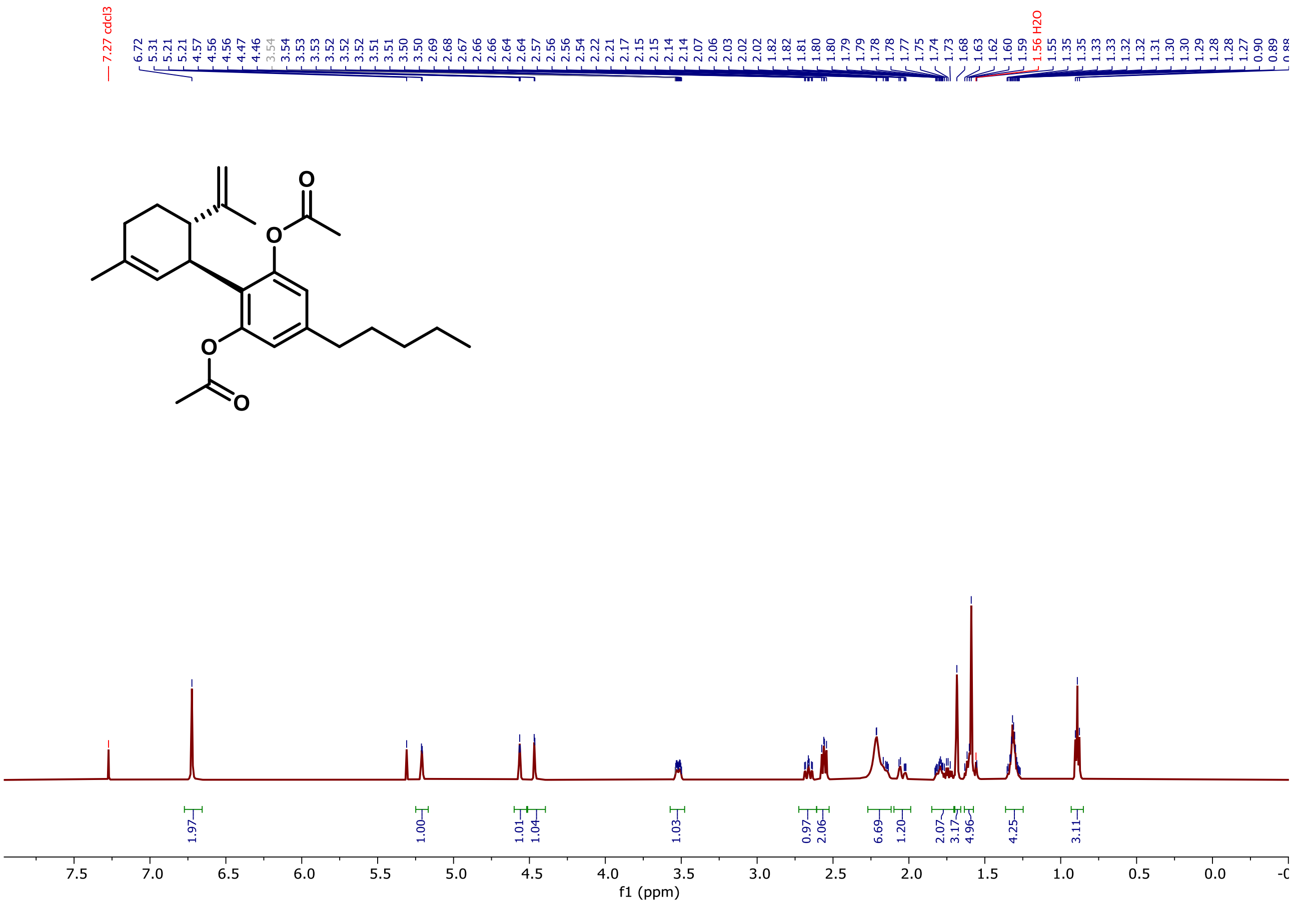

Figure S3: ^{1}H NMR of diacetyl cannabidiol.

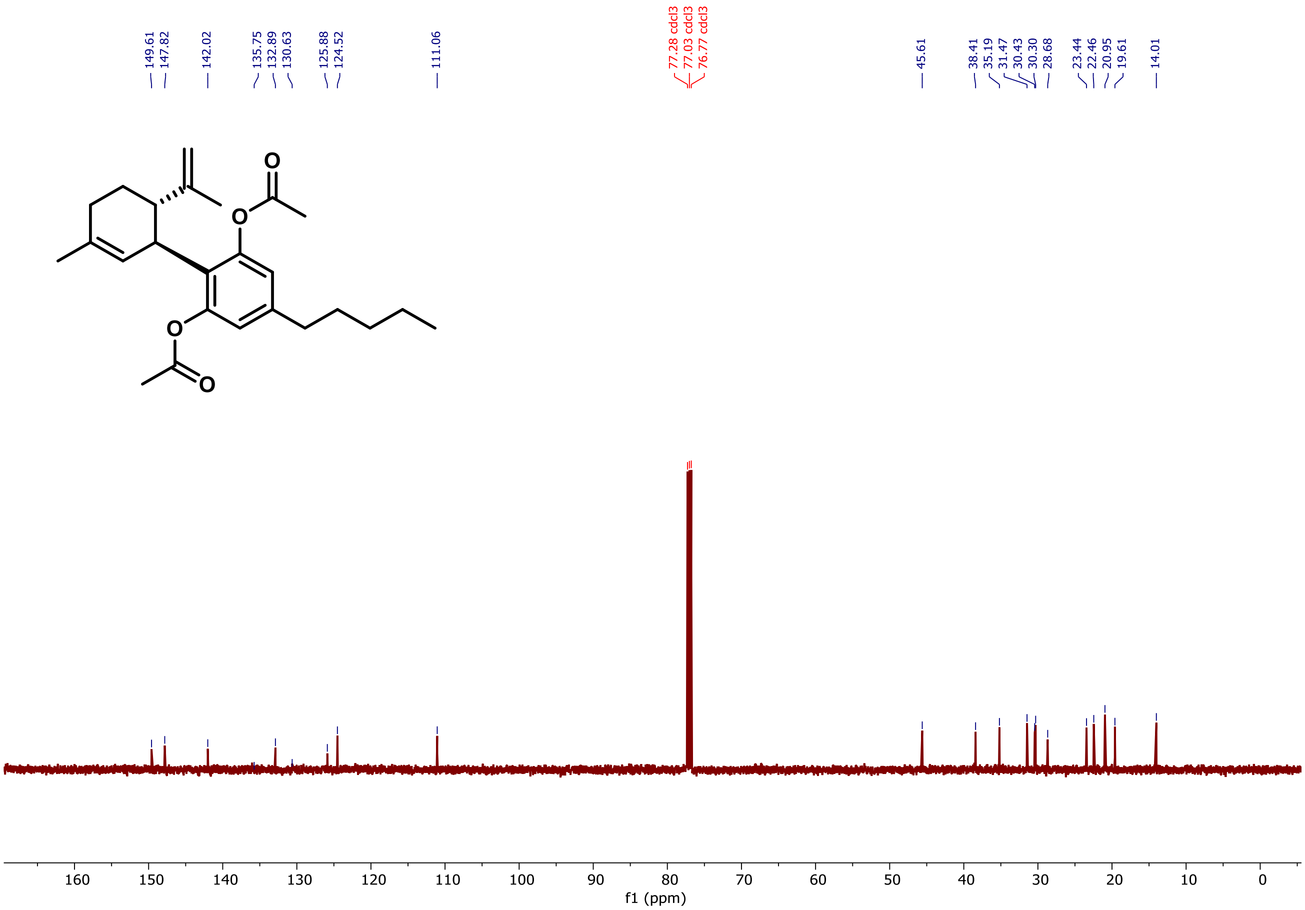

Figure S4: ^{13}C NMR of diacetyl cannabidiol.

S2. Mass spectrometry

All mass spectrograms were collected with a Thermo Fisher Scientific Q Exactive Plus mass spectrometer with Exactive Tune Software. Ionization was done in ESI Source in positive polarity only. A Hamilton syringe mounted to a syringe pump was used for continuous injection of samples at a 1 mM concentration (CBD in DMSO and CBD in EtOH, both kept for a month) dispensed at a flow rate of 10 μL/min. Each sample ion mass spectra were imported using Xcaliber 4.0 and the relative ion counts of the CBD and CBD-Q normalized and relative abundances calculated.

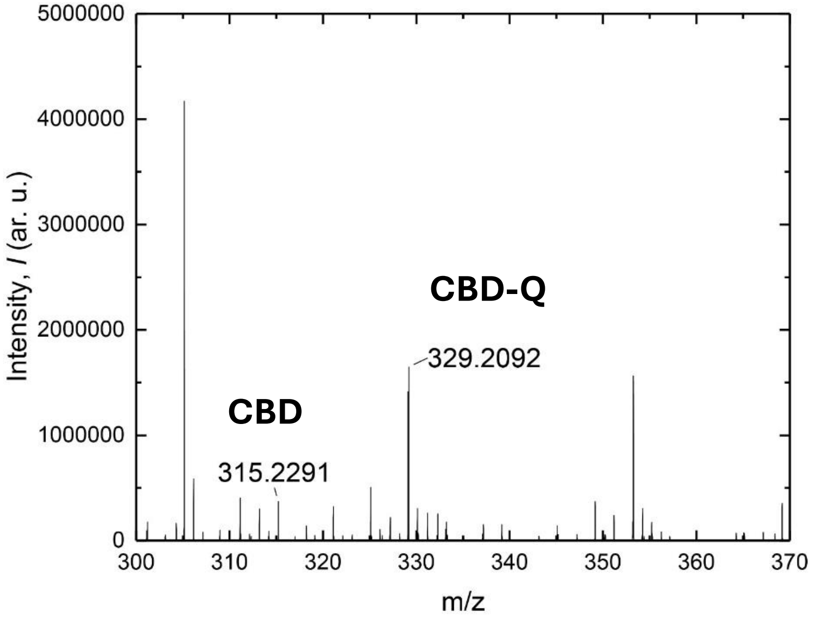

Figure S5. Ion spectrum (MS/MS) of CBD in EtOH stored for one month. Observed masses correspond to CBD-Q (*m/z* 329.2092), and CBD (*m/z* 315.2291).

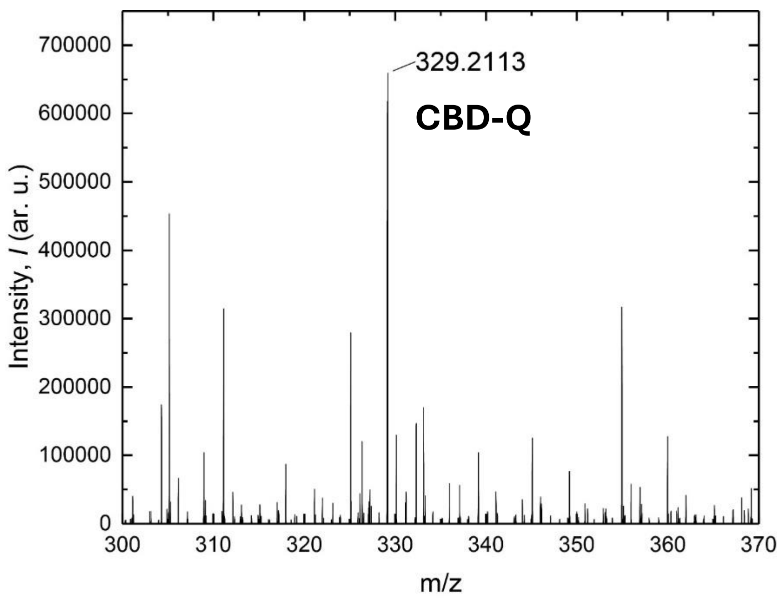

Figure S6. Ion Spectrum (MS/MS) of CBD in EtOH stored for one month. Observed mass is CBD-Q (*m/z* 329.2092). No unoxidized CBD was detected.

S3. Cyclic Voltammetry Procedure

In a borosilicate scintillation cell, tetrabutylammonium hexafluorophosphate supporting electrolyte (NBu_4PF_6) solution was added (5 mL, 0.1 M in CH_3CN). The glassy carbon working electrode, platinum counter electrode, and platinum pseudo reference electrode were positioned in the cell with ~0.5 cm of the electrode tips submerged in the electrolyte solution. CBD (0.5 mmol) was added to the cell and stirred until the solution was homogenous. A CV spectrum was collected. All spectra were collected with Gamry Instruments Framework, version 6.25, build 3318. All spectral analyses were performed with Gamry Echem Analyst, version 6.25.

Cyclic Voltammetry Parameters

E_{initial} (V): 0.5 vs. E_{ref}

Scan limit 1 (V): 3 vs. E_{ref}

Scan limit 2 (V): 0.4 vs. E_{ref}

E_{final} (V): 0.4 vs. E_{ref}

Scan rate (mV/s): 200

Electrode area (cm^2): 1

Equilibration time (s): 0

I/E range mode: auto *I/E* range

Maximum current (mA): 5

Cycles (#): 3

Open circuit (V): 0

Sampling mode: Noise rej

The oxidation potential of CBD dry and purged: 1700 V and 645 mA

The oxidation potential of CBD: 1600 V and 1200 mA

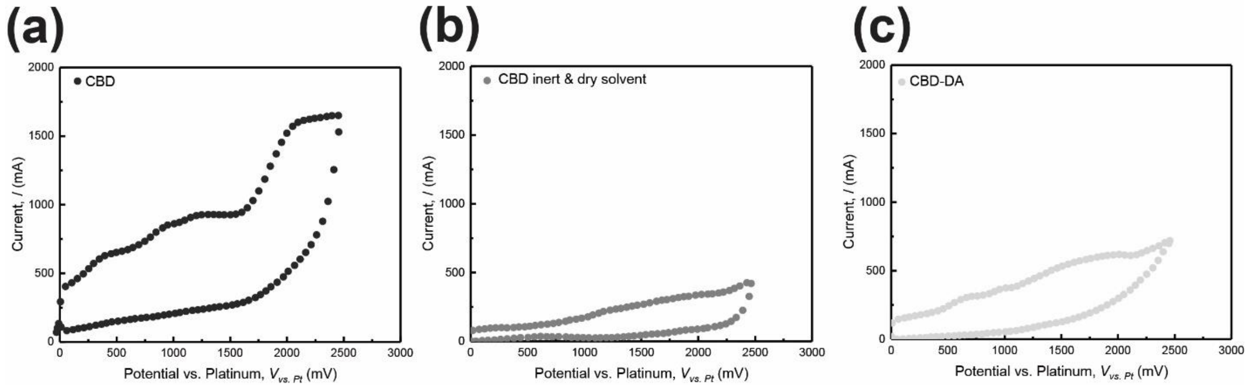

Figure S7. Cyclic voltammogram oxidation segment of **(a)** CBD in commercial non-dried ACN, **(b)** CBD in inert conditions and in dry ACN, **(c)** CBD-DA in commercial non-dried ACN.

In Figure S7, we present the three cyclic voltammogram oxidation segments of CBD and CBD-DA that were used to create Figure 1b in the manuscript. The CBD in commercial non-dried ACN represents CBD in an oxygen-rich environment, whereas the CBD in inert conditions and dissolved in dry ACN represents an oxygen-poor environment. Here, we demonstrate CBD has an oxidation potential only in an oxygen-rich environment, whereas, in the same environment, CBD-DA has no oxidation potential.

S4. Cell Culture

Expanding HUVECs

Human umbilical vein endothelial cells (HUVECs, Angio-Proteomie cAP-0001) of pass 3, 4, 7, 9 were expanded on 100 mm tissue-culture-treated polystyrene dishes (Corning® 430167) coated with 10 µL/mL fibronectin (FN, Corning® 356008) within a standard cell incubator at 37 ºC, 5% CO_2, and 95% humidity until cells were about 70–80% confluent. The HUVECs were fed every other day with the EGM™-2 with BulletKit™ from Lonza (CC-3162), where the media kit already contains about 2% FBS. These four batches of endothelial cells were used for four different external replicate experiments.

Toxicity Protocol

The day before the experiment, two 48-well tissue culture-treated polystyrene plates were coated with 10 µL/mL fibronectin (Corning® 354008). To do this, we diluted the stock 1 mg/mL fibronectin with PBS (Gibco™ 70011044) to yield a working concentration of 10 µg/mL. We added 200 µL of the 10 µg/mL fibronectin to each well and then placed in the plate in the cell incubator overnight.

On the day of the experiment, cells were stained with a nuclear stain (Hoechst, ThermoFisher 62249) and a live/dead viability/cytotoxicity kit (Invitrogen L3224), which contained a live cell stain, calcein AM, and a dead cell stain, ethidium homodimer-1. Stock concentrations of stains were as follows: 20 mM Hoechst, 1 mM calcein AM, and 2 mM ethidium homodimer-1. In an aliquot containing 8 mL EGM™-2, 2 µL of each stain was added, then the whole solution vortexed. The final working concentration of each strain was calculated to be 5 µM Hoechst, 0.25 µM calcein AM, and 0.5 µM ethidium homodimer-1. The cells were then rinsed with PBS (Gibco™ 70011044),

loaded with the stain-media solution, and placed in the cell incubator for 20 minutes. After 20 minutes, cells were checked for fluorescence and then prepared for passage.

Next, HUVECs were seeded onto the 48-well plates at a seeding density of 10,000 cells per cm^2 using standard cell passage protocols. Cells were allowed 3 hrs to settle, and in the meantime, fresh aliquots of the CBD analogs were dissolved to 5 mM stock concentrations in EtOH or DMSO.

We used crystalline CBD sourced from Tree House Hemp, whereas cannabidiol quinone and cannabidiol diacetate were both synthesized in lab with their purities confirmed using mass spectrometry (S1. Synthesis Procedures). Visually, CBD appeared to be a white crystalline powder whereas the CBD quinone and CBD diacetate were both in liquid form with a brown-orange or clear color, respectively. Two vials of each analog were prepped. The crystalline CBD, CBD-Q, and CBD-DA were then dissolved in ethanol (Fisher Scientific BP2818-500) or dimethyl sulfoxide (SIGMA Life Science D2650-100ML) to a stock concentration of 5 mM, then filtered using a 0.1 µm Millex™ PVDF filter (MilliporeSigma™ SLVVR33RS). These 5 mM stock concentrations were diluted further in EGM™-2 to their final working concentrations of 1, 6, or 10 µM to create the loaded media.

After 3 hours of allowing cells to settle, HUVECs were then rinsed with PBS, and each well was fed 300 µL of treated media. Then, the HUVECs were placed in a cell incubator for 24 hours. During the entirety of the experiment, all chemical and biological samples were stored and handled in the dark to the best of our ability (*i.e.*, lights in biohood off, chemical samples covered in aluminum foil).

Imaging

After 24 hours, cells were rinsed with PBS and fed a fresh batch of treated media. Next, a widefield epifluorescent inverted microscope (Nikon T2000EU) was used to image cells. Each well was imaged at the center of the well with a 10x magnification objective, 2s exposure, using the microscope's software, Micro-Manager 2.0.0.

The Hoechst stain was excited using a UV light source with 360 ± 40 nm excitation and emission was filtered using a UV Nikon filter (UV-2E/C). The calcein AM stain was excited with blue light with 480 nm ± 30 nm emission and filtered using a blue Nikon filter (B-2E/C). Ethidium homodimer-1 stain was excited using a green light source with 540 nm ± 25 nm emission filtered using a green Nikon filter (G-2E/C).

Each stain was imaged individually as a "slice" at the same focal plane. The three images were compiled together and saved as one image using Micro-Manager 2.0.0. This image was considered the "raw" image.

S5. Quantitative analysis of cytotoxicity

For further quantification, we created a counting cells macro to count the cells in each slice of our raw images using the software FIJI ImageJ2. At 10x magnification, for our microscope, 1 pixel was 0.34 µm.

The first part of our counting cells macro converted 16-bit images to 8-bit, subtracted the background, set the threshold, and converted the images to binary. We found that when keeping the images as 16-bit, FIJI had difficulty removing the background for an accurate cell count. Next, a median of radius = 20 pixels was set to eliminate small debris smaller than 7 µm, smaller than

the known HUVEC radii of, on average, greater than 20 µm. The analyze particle function had a size exclusion set to 50-infinity pixels (17 µm and larger), around the average known HUVEC diameters. Processed images were visually verified to ensure proper cell count. The resulting *.csv files generated from the analyze particles function were saved automatically and considered our raw *.csv data files. Each raw *.csv file contained all our internal replicates (n = 3) for each experiment date. Eventually, these internal replicates were averaged for each condition in each experiment using Microsoft Excel to generate the external replicates (N = 3 or 4).

For visualization, raw *.csv files from FIJI were then compiled into another data sheet, where calculated averages for the calcein AM stain were compiled. Hoechst stain analysis led to considerable variability, as it stains DNA content of both live and dead cells alike, masking toxicity results. For higher concentrations of CBD analogs, there were higher rates of cell toxicity, resulting in extremely low dead cell attachment, making the ethidium homodimer-1 results unreliable for toxicity quantification. Therefore, the calcein AM stain contained the most information regarding the state of the HUVECs upon exposure to CBD, whereas other slices were variable depending on cell attachment, which could be affected by PBS rinsing.

A Python script was used to graph boxplots, and the significance was added to the plots using Affinity Publisher 2.

All raw data, excel sheets, FIJI macros, and Python scripts are provided as supplemental materials. The following is the pipeline used to generate the graphs, and a guide to the items provided in the supplemental materials:

1. Raw images from fluorescence microscopy

2. Fiji Macro to analyze the raw images: “FijiMacro_countingCells_20240503.ijm”

3. The 4 resulting Fiji Excel Sheet per experimental replicate: “raw” + “YYYYMMDD” + “Summary.csv”

4. Compiled Excel Sheet with all raw data: “compiled_raw.csv”

5. Excel Sheet to specifically average Calcein data: “averagedCalcein_forPython.csv”

6. Excel Sheet with significance calculations: “calcein_Statistics.xlsx”

5. Python script to generate Figure 1, compiled using Jupyter Notebook Version 6.4.12, “averaged_Concentration_Boxplots_20230605” + “.py” or “.ipynb”

6. Used Affinity Publisher 2 to add significance bars.

We used this data analysis pipeline to produce graphs of the HUVEC viability after 24-hour exposure to CBD, CBD-Q, and CBD-DA at 1, 6, or 10 µM in either (a) DMSO or (b) EtOH. In the manuscript, we only presented the 1 and 10 µM conditions. Below, we present a few more supplementary graphs. The first, is a graph comparing the controls, where we quantified HUVEC after 24-hr exposure to: only EGM-2, 0.2% (v/v) EtOH in EGM-2 media, and 0.2% (v/v) DMSO in EGM-2 (Figure S8).

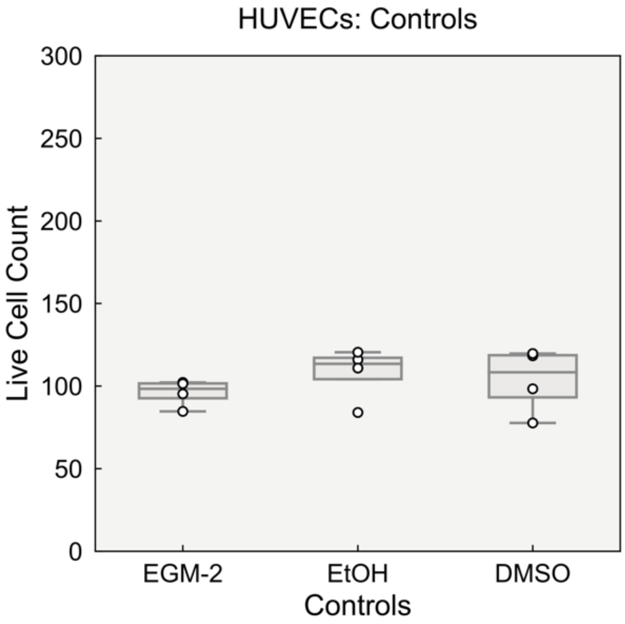

Figure S8: HUVEC viability after 24-hr exposure to EGM-2, 0.2% EtOH in EGM-2, and 0.2% DMSO in EGM-2. Boxplots include the aver-aged cell counts of each external replicate, represented by the data points, and whiskers representing the upper and lower quartile.

We mixed 0.2% of EtOH or DMSO into EGM-2 because to yield our maximum 10 μM concentration condition, we mixed 0.2% (v/v) of the CBD analog and vehicle (EtOH or DMSO) into EGM-2. In Figure S8, we observed that at this maximum 0.2% (v/v) EtOH or DMSO in EGM-2, the HUVEC viability was very similar to the cells exposed to their standard EGM-2 media. Therefore, we concluded that vehicle alone (DMSO or EtOH in EGM-2) did not contribute to the observed cytotoxic effects of CBD. Our 3-way ANOVA analysis also suggests that there is no relation between vehicle type (DMSO, EtOH) to CBD analog (CBD, CBD-Q, CBD-DA) or analog concentration (1, 6, 10 μM) that significantly impact HUVEC viability (SI Table 2).

In SI Figure S9, we also include the quantification of HUVEC viability after 24-hr exposure to CBD, CBD-Q, and CBD-DA at 6 μM in DMSO or EtOH as a vehicle. This 6 μM concentration was chosen because literature had reported 6 μM to be a critical concentration where CBD appeared to increase cell metabolic activity (Solinas 2012, Böckmann 2020).

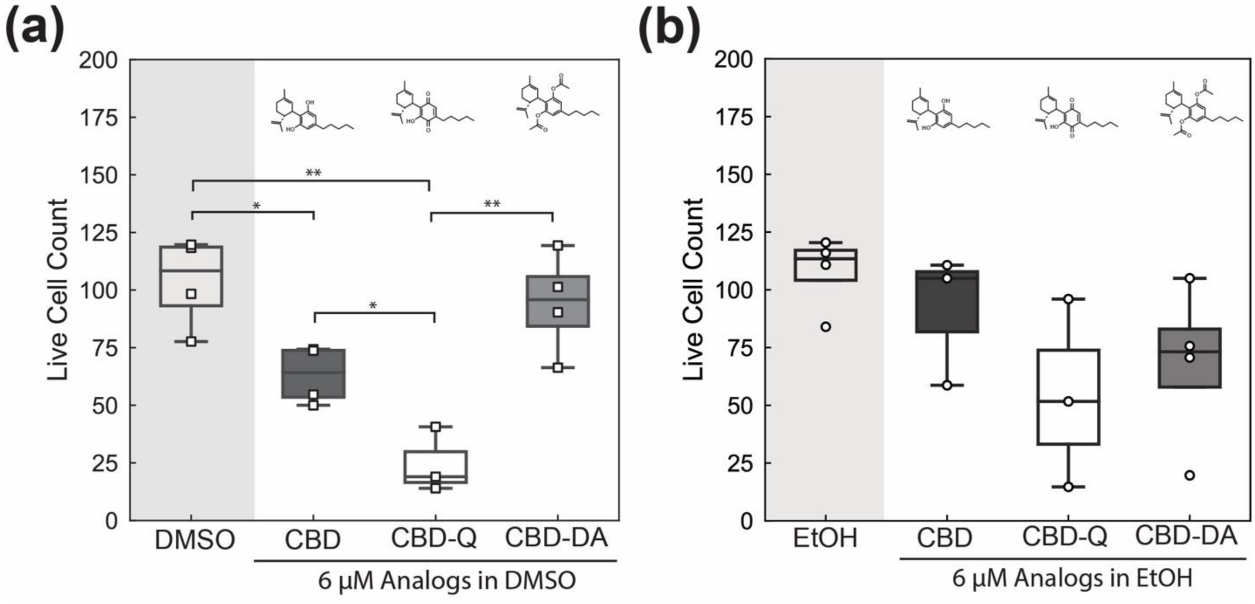

Figure S9: HUVEC viability after 24-hr exposure to CBD, CBD-Q, and CBD-DA at 6 μM in (a) DMSO as a vehicle and (b) EtOH as a vehicle. Boxplots include the averaged cell counts of each external replicate, represented by the data points, and whiskers representing the upper and lower quartile.

S6. Statistical analysis

After the averages of the internal replicates, n=3, were taken to yield the values of our external replicates, N=3,4, we used Microsoft Excel to conduct an *f*-test between all possible paired combinations of experimental conditions. If the resulting f-test value was less than 0.05, the pair was deemed to have significantly different variances and thus considered a two-sample unequal variance for the *t*-test calculation. If the value were greater than 0.05, then within the *t*-test calculation, the pair would be considered to have a two-sample equal variance.

Afterward, a *t*-test was conducted using Microsoft Excel. Using the averages of the internal replicates, a two-tailed *t*-test was performed. The type (equal or unequal variance) depended on whether the *f*-test yielded equal or unequal variances between the two samples tested. Finally, Microsoft Excel was also used to calculate the standard deviation of each internal replicate, which was then used to calculate the standard error of the mean (SEM).

Next, we conducted an ANOVA test to find differences between our indepentent variables. Here the ANOVA test assumes the dataset to have (1) equal sample sizes and (2) equal variance between conditions. For our data, we do not have different sample sizes therefore claim (1) does not hold true. We then conducted a Levene's Test of Equality of Error Variances to verify our dataset has equal variance. Indeed, Levene's Test showed significance values were all greater than 0.05, suggesting our dataset has equal variance (*Table 1*). Given that one of the two assumptions for the ANOVA test were true, we decided to conducted a 3-way ANOVA.

Table S1*:* Levene's Test of Equality of Error Variances. Table generated using IBM SPSS Version 29.0.2.0 (20).

Levene's Test of Equality of Error Variances[a,b]

		Levene Statistic	df1	df2	Sig.
CalceinCount	Based on Mean	1.308	17	45	.231
	Based on Median	.633	17	45	.847
	Based on Median and with adjusted df	.633	17	24.025	.833
	Based on trimmed mean	1.233	17	45	.280

Tests the null hypothesis that the error variance of the dependent variable is equal across groups.

a. Dependent variable: CalceinCount

b. Design: Intercept + drugType + drugVehicle + drugConcentration + drugType * drugVehicle + drugType * drugConcentration + drugVehicle * drugConcentration + drugType * drugVehicle * drugConcentration

The 3-way ANOVA test was conducted using the statiscical softwar: IBM SPSS Version 29.0.2.0 (20). Our tabulated values calculated in Microsoft Excel were then imported into IBM SPSS, consisting of three independent variables: "drugType" (CBD, CBD-Q, CBD-DA), "drugVehicle" (EtOH or DMSO), and "drugConcentration" (1, 6, 10 μM). The test considered how these three independent variables and their relation to each other, impacted our dependent variable: HUVEC viability (quantitative live cell counts, calculated from our calcein AM fluorescent staining) (*Table 2).*

Table S2: *3-Way ANOVA analysis table generated by IBM SPSS, comparing impact of drug type, vehicle, and concentration to HUVEC viability.*

Tests of Between-Subjects Effects

Dependent Variable: CalceinCount

Source	Type III Sum of Squares	df	Mean Square	F	Sig.	Partial Eta Squared
Corrected Model	34088.563[a]	17	2005.210	4.336	<.001	.621
Intercept	277354.568	1	277354.568	599.698	<.001	.930
drugType	11022.234	2	5511.117	11.916	<.001	.346
drugVehicle	434.298	1	434.298	.939	.338	.020
drugConcentration	11147.514	2	5573.757	12.052	<.001	.349
drugType * drugVehicle	545.187	2	272.594	.589	.559	.026
drugType * drugConcentration	8659.712	4	2164.928	4.681	.003	.294
drugVehicle * drugConcentration	201.527	2	100.764	.218	.805	.010
drugType * drugVehicle * drugConcentration	4364.566	4	1091.141	2.359	.068	.173
Error	20812.065	45	462.490			
Total	349987.444	63				
Corrected Total	54900.628	62				

a. R Squared = .621 (Adjusted R Squared = .478)

The 3-way ANOVA test indicates that drug concentration (1, 6, 10 μM) and drug type (CBD, CBD-Q, and CBD-DA) significantly impact HUVEC viability (p-value < 0.001). Additionally, when considering the paired relationship between drug type and drug concentration we also find our results to significantly impact the cell's viability (p-value < 0.003). However, when using a different drug vehicle (such as EtOH or DMSO) it does not seem to significantly impact the cell's viability (p-value of 0.338). Lastly, the drug type and the drug's concentration did not significantly impact the HUVECs viability (p-value > 0.05) when paired with the drug vehicle. Here we show that our results from the 3-way ANOVA agree with the student t-test results presented in Fig. 2 and Fig. 3, where we highlight the significance of drug type and drug concentration in resulting cell viability results.
